# Congenital tuberculosis (TB) use of second-line medication and therapeutic drug monitoring

**DOI:** 10.1515/crpm-2022-0019

**Published:** 2024-05-31

**Authors:** Haram A. Abdelmajid, Ghassan M.A. Mustafa, Antonia Fernandez, Susannah D. Franco, Americo E. Esquibies, Gloria B. Valencia, Stephan A. Kohlhoff

**Affiliations:** Division of Pediatrics Infectious Diseases, State University of New York (SUNY), Downstate Medical Center, New York, USA; Division of Neonatology, State University of New York (SUNY), Downstate Medical Center, New York, USA; Department of Pharmacy, State University of New York (SUNY), Downstate Medical Center, New York, USA; Division of Pediatric Pulmonology, State University of New York (SUNY), Downstate Medical Center, New York, USA

**Keywords:** mycobacteria, congenital, tuberculosis, drug-monitoring

## Abstract

**Objectives:**

Congenital tuberculosis (TB) is a rare complication of TB during pregnancy. Diagnosis and treatment are challenging, especially in premature infants due to limited data on drug dosing.

**Case presentation:**

A male infant was born at 33 weeks gestational age to a mother who was hospitalized with respiratory illness and diagnosed with TB after delivery. The diagnosis was based on the presence of granulomas and mycobacteria in the placenta, positive mycobacterium TB PCR in gastric aspirate, and growth of MTB from gastric aspirate and bronchoalveolar lavage cultures. Treatment started initially with isoniazid, rifampin, pyrazinamide, and ethambutol. The patient’s clinical course was complicated with ongoing respiratory distress and abdominal distension which raise the possibilities of drug resistance, but sensitivity was confirmed by PCR, or malabsorption of the medications due to concerns of abdominal TB. Isoniazid and rifampin doses needed to be adjusted due to low serum drug levels. Ethambutol was replaced by Levofloxacin, due to concerns about drug toxicity and unreliable therapeutic levels, but still there was no clinical improvement which raised the possibility of paradoxical reaction to the treatment, so we started the patient on prednisone for one month and tapered over two weeks. From age two months the patient showed progressive improvement and has by now completed 12 months of therapy with good clinical outcome.

**Conclusions:**

Therapeutic drug monitoring is important for adjusting medication dosage in premature infants due to limited experience in this age group.

## Introduction

Tuberculosis (TB) during pregnancy can lead to increased rates of spontaneous abortion, preterm birth, low birth weight, and increased neonatal mortality [1]. Congenital TB is a rare complication of intrauterine tuberculosis infection. It results from placental infection from either hematogenous spread through the umbilical vein, or through ingestion or aspiration of the amniotic fluid [1], [2], [3]. Unlike in adults, the primary focus of infection in congenital TB is usually the liver, with secondary spread to the lungs [1, 4], If not treated, congenital TB can be fatal in 50 % of cases [5]; but even with treatment, the mortality rate can still reach up to 22 % [5]. The rate of developing disseminated TB is higher in congenital TB patients than in other age groups [2, 5], because of their immature cell-mediated immunity [2]. The diagnosis of active TB during pregnancy is very important in reducing mortality and morbidity in the mother, the unborn fetus and the infant [2]. The diagnosis in newborns relies mainly on clinical suspicion and the presence of acid-fast bacilli in tissues and body fluids (i.e. gastric aspirate, bronchoalveolar lavage, and cerebrospinal fluid) by microscopy, polymerase chain reaction (PCR) or culture [3, 6], in addition to imaging [2, 4].

## Case presentation

The birth mother was 33 years old. This was her second pregnancy, with one previous birth (Gravida two Para one). She immigrated from Haiti in 2014 and visited Haiti in January 2020. She started prenatal care at nine weeks of gestation in March 2020, and didn’t report cough, fever, night sweats or weight loss. The QuantiFERON (IGRA) obtained during the first visit was positive, and HIV Ag/Ab Combo test was negative.

At 30 weeks gestation, she was admitted to the hospital with a three-day history of cough, but without symptoms typically associated with TB. Chest radiograph showed a calcified granuloma in the right hilum and a diffuse parenchymal process with partial vascular obstruction. The Chest Computed Tomographic (CT) scan showed scattered foci of ground glass opacities in the upper lungs bilaterally, and a right lower lobe punctate calcified granuloma. Induced sputum for acid fast bacilli (AFB) smear and culture were sent on hospital days, four, eight and 22. Smears were negative for AFB. Because of the negative smears Mycobacterium PCR was not requested. Tests for Severe Acute Respiratory Syndrome Corona Virus 2 (SARS-COV2) were negative.

The mother developed fever (39.7 °C oral) and chills on the first day of admission and remained febrile, with deterioration in her respiratory status in the next 48 h, requiring oxygen support. She continued to be febrile despite treatment with ceftriaxone 1 g per day to cover for suspected community acquired pneumonia, and azithromycin 500 mg per day to cover for atypical pneumonia (discontinued after three days because the patient did not respond), vancomycin 15 mg/kg every 12 h administered for seven days, and dexamethasone 6 mg/day for five days.

On the 22nd day of admission, she developed acute hypoxic respiratory failure and was admitted to the Medical Intensive Care Unit (MICU), another chest CT scan showed persistence of diffuse opacities involving all lung fields, more prominent than in the prior study. Following extensive rheumatology, infectious disease and pulmonology work up, the diagnosis of systemic sarcoidosis with pulmonary involvement was considered, and she was started on intravenous (IV) methylprednisolone 125 mg every 12 h for three days followed by 100 mg every 12 h for seven days. On admission day 30, at 33 weeks gestation labor was induced and a male infant was delivered by emergency cesarean section due category two fetal tracing.

After delivery, the mother continued to have persistent fever and developed headache, with little improvement in her respiratory status. On the seventh day post-delivery, alteration in her mental status was noted, a head CT scan showed non-obstructive hydrocephalus with bilateral hypodensities in parietal regions. A subsequent brain magnetic resonance imaging (MRI) was done and showed ring-enhancing lesions with restricted diffusion, hydrocephalus, and acute right posterior cerebral artery infarct. 12 days post-delivery the results of the placental pathology revealed multifocal necrotizing granulomatous villitis with Langhans-type multinucleated giant cells and micro abscesses. AFB special stains were consistent with *M. tuberculosis* infection. She started on anti-tuberculous medications but remained critically ill until she developed brain herniation and expired 30 days after delivery. Autopsy confirmed disseminated *M. tuberculosis* infection.

A male infant was delivered by emergency C-section due to category II tracing with an average weight for gestational age (2,155 g). APGAR scores were eight and nine at 1 and 5 min, respectively. At 7 min of life, he developed respiratory distress and low oxygen saturations for which he received continuous positive airway pressure (CPAP). The gross physical examination was normal. Initial chest X-ray was significant for mild diffuse granular airspace opacities suggestive of respiratory distress syndrome, he was weaned to room air within the first 24 h. Following resolution of initial respiratory symptoms, he was feeding well on room air without respiratory support.

After the results of the placental pathology became available on day 12 post-delivery, the infant was evaluated for congenital tuberculosis, and on day 15 anti-tuberculous treatment was initiated (isoniazid 10 mg/kg with pyridoxine supplementation, rifampin 15 mg/kg, pyrazinamide 35 mg/kg, and ethambutol 20 mg/kg). The gastric aspirate and bronchoalveolar lavage (BAL) were positive by PCR (GeneXpert, Cepheid) for *M. tuberculosis.* There was no *rpoB* mutation detected. All other tests including three gastric aspirates for AFB smear and culture taken at ages 12, 13 and 14 days, BAL at age 14 days for AFB smear and culture, and urine, and blood AFB smear, culture and PCR at age 12 days, were negative. The chest radiograph on day 12 showed persistent air-space opacities seen on day 1.

**Figure 1:**
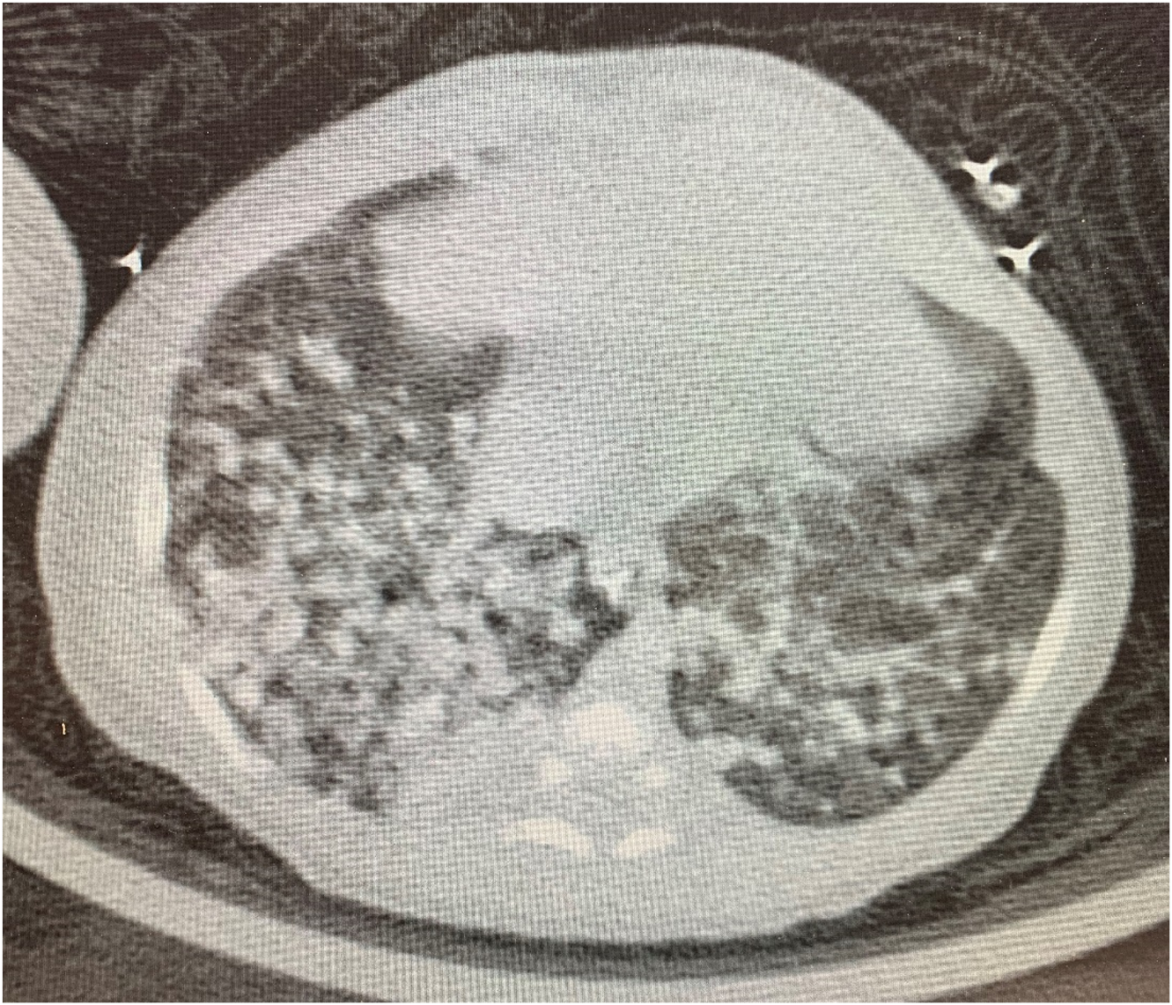
CT scan lungs and airways of the infant, showing diffuse nodular parenchymal opacities bilaterally, most significantly involving the right hemithorax September 2020.

At age 16 days, the baby developed tachycardia, and tachypnea, with requirement of non-invasive respiratory support via nasal CPAP and he was started on vancomycin 15 mg/kg/dose every 8 h, and piperacillin-tazobactam 80 mg/kg/dose every 6 h (dosage based on piperacillin component only) as broad coverage for suspected neonatal sepsis. He showed some improvement, and the antibiotics were continued for seven days. The chest radiograph obtained at age 16 days was significant for diffuse airspace opacities, and focal consolidation within the right lung. At 25 days of age, the baby developed abdominal distension which progressed to severe ileus. There was hepatosplenomegaly on examination. Abdominal TB was suspected, and vancomycin was restarted and meropenem was initiated. At age 28 days, he developed fever of 38.4 °C, associated with tachypnea and tachycardia. Amikacin was given as a second-line anti-tuberculous medication from age 28–40 days, without improvement, and subsequently discontinued. Because of failure to respond to antibiotic therapy, a chest CT was obtained on day 37 of age, which showed diffuse nodular parenchymal opacities bilaterally, most significantly involving the right hemithorax (Figure 1) consistent with pulmonary TB.

At age 45 days, the patient was started on prednisolone (2 mg/kg/day for one month and tapered over three weeks) assuming that the patient’s failure of response is due to a paradoxical reaction to anti-tuberculous medications.

MRI of the brain at 24 days old was unremarkable. A subsequent MRI at 47 days old was done due to episodes of staring, showed non-specific changes of corpus callosum that might be associated with TB. Neurological examination and electroencephalogram (EEG) were unremarkable. MRI at age 71 days showed resolution of the initial findings of concern.

Rifampin, isoniazid, pyrazinamide, and ethambutol doses were adjusted weekly according to the infant’s weight and drug concentrations (Table 1), and on the 50^th^ day of age - 36 days since the start of therapy, ethambutol (started at first as part of the standard regimen of TB treatment) was replaced with levofloxacin due to concerns about ethambutol eye toxicity as well as its unpredictable pharmacokinetics in a young infant. Because of uncertain pharmacokinetics of some of the anti-tuberculous drugs used and the fact that the patient did not show any radiological and clinical improvement after 5 weeks of therapy, an extension of the initiation phase of treatment from 2 to 4 months was decided.

**Table 1:** Therapeutic drug levels of Rifampin and Isoniazid and the corresponding doses.

Medication	Patient’s age, weeks	Dosage, mg/kg/day	Peak level µg/mL	Normal range µg/mL
Rifampin	8	15	1.5	5–27
	10	20	2.5	
	12	20	8.8	
Isoniazid	8	10	0.81	1–7
	10	15	2.5	

Peak level is measured 2 h after administration of the medication.

As shown in Table 1, the initial dose of rifampin was 15 mg/kg orally daily. Based on the 2 h (peak) therapeutic goal level of 8–24 μg/mL and his serum level of 1.5, the dose was increased to 20 mg/kg orally daily [7]. Three days later, the rifampin peak was 2.8 μg/mL, which was still subtherapeutic. However, on the day the peak was obtained, the patient had a large spit-up within 1 h of rifampin administration. Repeated episodes of spitting up and vomiting associated with rifampin administration was not improved by providing small volumes of feeds prior to administration or by altering the taste of the medication with cherry syrup [8]. Therefore, ondansetron 0.15 mg/kg was initiated, given 30 min prior to rifampin. A baseline electrocardiogram (EKG) was done to monitor QT because of concerns about cardiotoxicity associated with co-administration of ondansetron and levofloxacin. QT was normal, and the EKG was done monthly to monitor progress. The patient had no additional episodes of vomiting after ondansetron was started, and rifampin levels (on the same dose of 20 mg/kg) were determined to be therapeutic at 8.8 g/mL [7].

µDrug peak concentrations were obtained throughout the infant’s admission to guide dosing [9] to maximize pharmacological efficacy, using this formula:

$$I_{3}=\frac{C_{3h+0.6}}{D}$$

*C*_3h_=peak level (3 h after dose), *D*=dose, *I*_3_=inactivation index [9].

Dose adjustments were made based on pharmacokinetic calculations and reported usual dose ranges [7, 9]. Isoniazid was initially given at a dose of 10 mg/kg orally daily. While the initial peak reached was 2.5 μg/mL, the following peak was subtherapeutic at 0.81 μg/mL based on isoniazid three-hour (peak) goal of 1–6 μg/mL [7, 9]. Using the equation above, the resulted concentration of 0.81 μg/mL, and a goal concentration of 1.5 μg/mL, our patient’s inactivation index was calculated to be 0.14, and his dose was increased to 15 mg/kg orally daily. The subsequent isoniazid peak was therapeutic at 2.5 μg/mL [9]. Eventually, drug susceptibility was confirmed for isoniazid, rifampin, ethambutol, and pyrazinamide based on the mother’s sputum AFB culture obtained around the time of birth and results obtained at baby’s age of 65 days.

The patient continued to improve clinically with minimal changes in the chest X-ray, and was discharged home at four months of age with plans to continue four anti-tuberculous medications, isoniazid 15 mg/kg/day, rifampin 20 mg/kg/day, pyrazinamide 35 mg/kg/day, and levofloxacin 15 mg/kg/day, with dose adjustment according to weight at each visit for a total of four months, and continuation phase with isoniazid 15 mg/kg/day and rifampin 20 mg/kg/day of nine months. In September 2021, he completed 12 months of treatment, with complete clinical and radiological resolution and normal growth and development.

## Discussion

This case highlights the importance of early detection and treatment of TB during pregnancy in preventing maternal and neonatal morbidity and mortality. Congenital TB, although rare [3], is associated with a very high morbidity and lethality [5], but it is preventable by having a high index of clinical suspicion, and thorough evaluation of pregnant people, especially those who have underlying risk factors, or immigrated from or travelled to TB endemic countries as in the present case. The disease usually presents between one – 84 days of age with an average age of presentation being two to four weeks old [10]. The symptoms of congenital TB are mostly non-specific [10]. The infant can present with poor feeding, failure to thrive, respiratory distress, fever, hepatosplenomegaly, lethargy, or irritability [2, 10]. Although the chest radiograph for the infant presented here was abnormal from day one, he was asymptomatic until 2 weeks of age. It takes six – eight weeks for the culture to grow, which underscores the importance of early diagnosis and treatment of maternal TB infection to avoid a fatal outcome. In 1994, Cantwell, et al. proposed revised diagnostic criteria for congenital TB [10]: “proved TB lesions in the infant plus at least one of the following: (1) lesions in the first week of life; (2) a primary hepatic complex or caseating hepatic granuloma; (3) tuberculosis infection of the placenta or maternal genital tract; (4) exclusion of the possibility of postnatal transmission by thorough investigation of contacts.” This patient met these criteria with an abnormal chest radiograph on day one and AFB in the placenta.

One of the major challenges that may have impacted the persistence of the infant’s infection was the difficulty in achieving stable therapeutic concentrations of anti-mycobacterial drugs and the lack of guidance and evidence-based practice due to the paucity of literature for dosing in infants. As a result, we had to rely on established adult therapeutic goals to guide dosing at the initiation of treatment, and then make adjustments empirically to reach our goal peak concentrations, but with greatly delayed timing because of how long it would take to receive tests results. There are no published studies reporting drug levels in lung tissue of premature infants, which is relevant to rationalizing a response to therapy in that patient population. The coordination of oral and nasogastric feeds and drug administration, as well as rifampin-associated vomiting, also considerably contributed to the difficulty and time in achieving stable therapeutic concentrations. Of note, administering rifampin via nasogastric tube instead of by mouth did not improve vomiting, and only with the addition of ondansetron the vomiting resolved. Creating an hourly schedule to appropriately spread feeding and medication administration times was most useful in improving the consistency of medication administration, and maintenance of therapeutic anti-tuberculosis medication levels. We also timed rifampin and isoniazid doses so that their respective 2 and 3 h concentrations could be measured from a single blood draw.

The pathogenesis of TB during pregnancy is not different from non-pregnant people, except when it affects the genital tract, or rarely, the placenta [2]. In our case, it appears placental infection led to direct dissemination to the fetus. Diagnosis of TB during pregnancy is challenging and might be missed or delayed due to the similarities of pregnancy symptoms with the symptoms of TB [2]. Otherwise, diagnostic tests are similar to nonpregnant people, including the tuberculin skin test, IGRA, radiographic evaluation, and sputum detection of AFB by microscopy, PCR, and culture. Although the mother had had some symptoms consistent with TB, a positive IGRA, and an abnormal chest radiograph, her initial sputum smear for AFB was negative, and so PCR was not performed initially and she was not started on anti-tuberculous medication as a result, as per standard practice given those negative findings on the sputum smear. The use of PCR testing on the maternal respiratory sample could potentially have resulted in a timelier diagnosis and treatment. Furthermore, she received one week of high dose steroids on the basis of clinical suspicion of sarcoidosis. Looking back, all these factors together may have contributed to the dissemination of TB and the resulting maternal mortality, burden of infection and prolonged morbidity of the newborn.

In summary, recognition of TB during pregnancy remains a challenge, yet is critical to the prevention of congenital TB. Furthermore, medical management of congenital TB in premature critically ill newborns may require utilization of therapeutic drug monitoring and use of second-line agents. More research and data on TB drug pharmacokinetics in infants are needed.
